# Potential Health Claims of Durum and Bread Wheat Flours as Functional Ingredients

**DOI:** 10.3390/nu12020504

**Published:** 2020-02-17

**Authors:** María Ciudad-Mulero, Lillian Barros, Ângela Fernandes, Isabel C.F.R. Ferreira, Mª Jesús Callejo, Mª Cruz Matallana-González, Virginia Fernández-Ruiz, Patricia Morales, José M. Carrillo

**Affiliations:** 1 Department of Nutrition and Food Science, Pharmacy Faculty, Complutense University of Madrid (UCM), Plaza Ramón y Cajal, s/n, E-28040 Madrid, Spain; mariaciudad@ucm.es (M.C.-M.); mcmatall@ucm.es (M.C.M.-G.); vfernand@ucm.es (V.F.-R.); 2 Centro de Investigação de Montanha (CIMO), Instituto Politécnico de Bragança, Campus de Santa Apolónia, 5300-253 Bragança, Portugal; lillian@ipb.pt (L.B.);; 3 Unidad de Genética, Departamento de Biotecnología y Biología General, E.T.S.I.A.A.B., Universidad Politécnica de Madrid, Ciudad Universitaria s/n, 28040 Madrid, Spain; mj.callejo@upm.es (M.J.C.); josem.carrillo@upm.es (J.M.C.)

**Keywords:** wheat fractions, EFSA nutritional and health claims, dietary fiber, arabinoxylans, tocopherols, phenolic content, antioxidant activity

## Abstract

Wheat is an important cereal with a key role in human nutrition. In this study, dietary fiber (DF) and arabinoxylans of different durum (*Triticum turgidum* ssp. *Durum* L.) and bread (*Triticum aestivum* L.) wheat flours were analyzed in order to point out their potential nutritional and health claims allege according to the current European regulation (Regulation (EU) No 432/2012). Moreover, other bioactive compounds (phenolics and tocopherols) were quantified as a first approach to their phytochemical composition in the analyzed wheat varieties. DF was analyzed following AOAC enzymatic-gravimetric methods; arabinoxylans and total phenols were quantified by colorimetric methods; tocopherols were determined by HPLC; antioxidant activity was evaluated using three different in vitro assays. Insoluble DF was the prevailing fraction in all analyzed samples. Water extractable arabinoxylans were higher in durum wheat flours. Whole flours contained higher total phenolics compounds. Alpha-tocopherol was the major isoform. Whole flours showed higher antioxidant properties. According to the obtained results, it is possible to allege all approved health claims referred to wheat, since all analyzed samples, especially whole flour and bran fraction, showed potential health benefits, as functional ingredients or functional foods, related with their phytochemical composition.

## 1. Introduction

Wheat is an important cereal worldwide that plays an outstanding role in human nutrition. It is used in a wide range of food products such as bread, cookies, cakes and noodles [1,2]. Durum wheat flours (*Triticum turgidum* ssp. *Durum* L.) are technologically more suitable for good-quality pasta production, contributing to the final bright yellow color, its capacity to retain after cooking firmness, and it is resistant to surface disintegration and stickiness [3], whereas, bread wheat flours (*Triticum aestivum* L.) are the most widely used cereals for bakery products elaboration, e.g., bread [4].

Most of the bioactive compounds in wheat are located in the bran fraction and especially in the aleurone layer [5]. Regarding the different wheat flours fractions, those of greatest interest from the technological and nutritional point of view are whole wheat flour (also known as whole-grain flour) and white wheat flour (also known as refined flour). Whole wheat flour, which is rich in resistant carbohydrates (mainly dietary fibers), vitamins, phenolic acids and minerals (like Fe and Zn), results of grinding whole wheat grains or combining the different milling fractions of the clean grain. White flours, consist of the flour made only using grain endosperm (during the milling process it is devoid of germ, bran and aleurone layer) and therefore have a higher aptitude for leavening and are preferred to whole meal flours for the manufacture of certain baked, leavened products (special occasion cakes), etc. [4].

It is known, that whole grain consumption has been associated with reduced risk of developing chronic diseases, including type 2 diabetes, obesity, cardiovascular diseases, and cancer. This health benefit of whole grain consumption may be due to different phytochemicals content (phenolic compounds, which containing one or more aromatic rings and one or more hydroxyl groups, and vitamin E, amongst others) [6].

Due to growing evidence of the health benefits of whole grain intake, the aim of this study was to compare different wheat flours (whole grain flour, white flour and bran fraction) obtained from different wheat varieties (Endural and Aldura, corresponding to durum wheat; and Cajeme and Marius, corresponding to bread wheat), as part of a genetic improvement project. The genetic improvement of the nutritional quality in different wheat flours was focused on the analysis of the composition and variability of bioactive compounds (dietary fiber, arabinoxylans, phenolic compounds) and the evaluation of the antioxidant activity in the studied samples. Therefore, the main purpose of the present study was to point out the potential nutritional and health claims that can be highlighted and mentioned in the food labeling of the wheat analyzed varieties, based on their composition and the current European regulation, in order to valorize these interesting wheat varieties traditionally consumed in Spain. Therefore, it was necessary to characterize the phytochemical composition of the mentioned wheat varieties in order to evaluate their functional properties as well as the possibility of the use different approved heath claims in accordance with the current regulation. Moreover, other bioactive compounds (e.g., phenolics and tocopherols) were quantified as a first approach to their composition in the analyzed wheat varieties.

## 2. Material and Methods

### 2.1. Wheat Samples

Whole grain flours, white flours and bran fractions from different bread wheat (*Triticum aestivum* L., Cajeme and Marius varieties) and durum wheat (*Triticum turgidum* ssp. *Durum* L., Endural and Aldura varieties) varieties were analyzed. The wheat grains were harvested in two different and consecutive years and processed in Department of Biotecnología of the Escuela Técnica Superior de Ingeniería Agronómica, Alimentaria y de Biosistemas, Universidad Politécnica de Madrid, Spain (40°26′47.36″ N, 3°44′21.00″ W). For dietary fiber and arabinoxylans, two different harvested samples were analyzed for each wheat variety (n = 6). However, for phenolics, tocopherols and antioxidant capacity, each wheat variety was harvested in two different and consecutive years and were mixed into a unique and representative sample, avoiding the possible agronomical and environmental interferences (n = 3).

### 2.2. Milling Process

The whole grain flour was obtained using a Tekator Mill equipment and sieve with 1mm of mesh. To obtain white flour, a Chopin CD1 mill was used in 4 stages (1 crushing and 3 compressions).

### 2.3. Analysis of Bioactive Compounds

#### 2.3.1. Total, Soluble and Insoluble Dietary Fiber

Total, soluble and insoluble dietary fiber were quantified according to AOAC enzymatic-gravimetric methods 993.19 and 991.42, respectively [7,8]. In total, 0.3 g of the sample was incubated at 100 °C for 15 min with shaking in 50 mL of phosphate buffer (pH = 6.0 ± 0.2) solution containing 0.1 mL of α-amylase. The pH was adjusted (pH = 7.5 ± 0.2), by the addition of 10 mL of 0.275 N NaOH (5 mg of protease was also added). After incubation at 60 °C for 30 min with shaking, the pH was adjusted (pH = 4–4.6) by the addition of 10 mL of 0.325 N HCl (0.1 mL of amyloglucosidase was also added). The samples were incubated for 30 min at 60 °C with shaking. The mixture was filtered (insoluble fiber) and the filtrate was collected in a 500 mL Erlenmeyer flask. After precipitation by the addition of 400 mL of ethanol it was filtered again (soluble fiber). Duplicate samples are always processed, in order to subtract proteins and ashes for the calculation of the total dietary fiber (TDF) content. Dietary fiber (total, soluble and insoluble) values are expressed as g/100 g dw flour.

In the case of bran fraction, total, soluble and insoluble dietary fiber were calculated by application of the following equation (Equation (1)):

(1) $$Bran\;concentration=\frac{Whole\;grain\;concentration-\left(White\;flour\;concentration\times Extraction\;rate\right)}{\left(1-Extraction\;rate\right)}$$

#### 2.3.2. Arabinoxylans

Arabinoxylans (TO-AX and WE-AX) were quantified following the colorimetric methods described by Ciudad-Mulero et al. (2018) [8]. In brief, samples (125 mg) were placed in a 50 mL graduated conical polypropylene tube and 25 mL of distilled water was added. The tubes were shaken for 30 min and after that, 0.5 mL of the suspension, which contained 2.5 mg of the sample, was placed into a stoppered reaction tube in order to determine the TO-AX content. The original sample suspension was centrifuged at 2500 rpm for 10 min (Universal 16 R, Genesys Instrumentacion, SL, Madrid, Spain) and after centrifugation, 0.5 mL of the supernatant was placed into an amber reaction tube, similar to the method above. The supernatant aliquot represented a 2.5 mg equivalent of the sample and it was used for the determination of WE-AX content. Two sample suspensions were made from each sample and two aliquots of 0.5 mL were removed from each sample suspension. In this way, four sample replicates resulted the TO-AX content (two from the first sample suspension and two from the second sample suspension).

For each centrifuged sample suspension, two aliquots (0.5 mL) of the supernatant were removed, resulting in four sample replicates for WE-AX content (two from the first sample suspension and two from the second sample suspension). 

Sample aliquots were collected, and 1.5 mL of distilled water was added (final volume was 2 mL). Arabinoxylans (TO-AX and WE-AX) were determined using a colorimetric method. A calibration curve was simultaneously prepared using a stock solution of 10 mg of D-(+)-xylose in 100 mL of distilled water. Then, triplicate standard samples were prepared using different concentrations of xylose (0.005–1 mg/mL). The values are expressed as g per 100 g dw flour.

In particular, arabinoxylans (TO-AX and WE-AX) content in bran fraction were calculated using the Equation (1).

#### 2.3.3. Tocopherols

Tocopherols were determined following a procedure described by Morales et al. (2015) [9] and Ciudad-Mulero et al. (2018) [8], using HPLC (Knauer Smartline system 1000, Berlin, Germany) coupled to a fluorescence detector (FP-2020; Jasco, Easton, MD, USA) programmed for excitation at 290 nm and emission at 330 nm. The chromatographic separation was performed using a Polyamide II (250 × 4.6 mm) normal phase column from YMC Waters (Japan) operating at 30 °C (7971 R Grace oven). The quantification was based on the fluorescence signal response (of each standard, using the internal standard (tocol) and calibration curves that were prepared using commercial standards of each compound. The results are expressed as mg per 100 g of flour (dw).

#### 2.3.4. Total Phenols Content

Total phenols content in the methanolic extract of the different samples was estimated by a colorimetric assay, based on procedures described by Wolfe et al. (2003) [10] with some modifications. 1 g of each sample was weighed and methanolic extracts were obtained by stirring samples with 40 mL of methanol at 25 °C for 1 h. The resultant extracts were filtered and the residue was then re-extracted with one additional portion of methanol. The combined methanol extracts were evaporated at 35 °C under reduced pressure (rotary evaporator Büchi R-210, Flawil, Switzerland), re-dissolved in methanol at a concentration of 5 mg/mL, and stored at 4 °C for further use [9]. Gallic acid was used as the calibration curve standard, and the reduction of Folin-Ciocalteu reagent by the samples is expressed as mg of Gallic acid equivalents (GAE) per g of extract.

#### 2.3.5. Evaluation of Antioxidant Activity

The in vitro antioxidant activity assays were performed following the previously described methodology by Morales et al. (2015) [9]. Methanol extract was further diluted to different concentrations to be used in the antioxidant activity assays. The in vitro antioxidant activity assays, DPPH radical-scavenging activity, reducing power and inhibition of β-carotene bleaching, were performed, using Trolox as positive control. In brief, DPPH radical-scavenging activity was evaluated using an ELX800 microplate Reader (BioTek Instruments, Inc.; Winooski, VT, USA) and calculated as a percentage of DPPH discoloration after 1 h of incubation with the antioxidant extract measuring the absorbance of the solution at 515 nm. The reducing power was evaluated by the capacity to reduce Fe^3+^ to Fe^2+^, measuring the absorbance at 690 nm the microplate reader mentioned above. Inhibition of β-carotene bleaching was evaluated through the β-carotene/linoleate assay; the neutralisation of linoleate free radicals avoids β-carotene bleaching. Within β-carotene bleaching assay, measurement is done at 470 nm; at this wavelength other carotenoids in the sample might be a bias for the results. The results of the antioxidant activity are expressed as EC_50_ values (mg/mL methanolic extract) [9].

### 2.4. Statistical Analysis

Mean ± standard deviations (SD) were determined using Statgraphics Plus 5.1 software to analyze data at the 95% confidence level. The data were statistically analyzed by analysis of variance (ANOVA), followed by Tukey test. The statistical significance level was set at *p* < 0.05.

## 3. Results

### 3.1. Dietary Fiber and Arabinoxylans

Dietary fiber fractions and arabinoxylans content in analyzed wheat flours are shown in Table 1. Regarding dietary fiber fraction, statistically significant differences between years in white flours were observed, while in whole grain flours and bran, the statistically significant differences were found between bread and durum varieties. Particularly, all the analyzed whole grain flours and bran from bread wheat varieties present significantly higher amounts of TDF (*p* < 0.05) than the corresponding fractions from durum wheat varieties.

Regarding durum wheat flour (*T. turgidum*, Endural and Aldura varieties), total dietary fiber (TDF) ranged from 5.4 to 7.0 g/100 g dw in white flours, from 13.1 to 17.0 g/100 g dw in whole grain flours and from 23.5 to 32.1 g/100 g dw in bran fraction. While, in the case of bread wheat samples (*T. aestivum*, Cajeme and Marius varieties), the TDF content was found between 4.7–6.9 g/100 g dw in white flours, 18.2–19.8 g/100 g dw in whole grain flours and between 46.1–51.6 g/100 g dw in bran fraction.

In all cases, the insoluble dietary fiber (IDF) was the prevalent fraction, representing up to 96% of TDF (as in the case of S-M#1-WF), while, as expected, soluble dietary fiber (SDF) was found in lower amounts (Table 1). 

In the analyzed flours, total arabinoxylans (TO-AX) content ranged from 4.2 to 7.0 g/100 g (dw) in white flours, 6.5 to 8.5 g/100 g (dw) in whole grain flours and 6.3 to 13.8 g/100 g (dw) in bran fraction. While, water-extractable arabinoxylans (WE-AX) content were found in lower amount, with values ranging from 0.3 to 1.0 g/100 g (dw) in white flours, 0.5 to 1.1 g/100 g (dw) in whole grain flours and 0.3 to 1.9 g/100 g (dw) in bran fraction (Table 1). The whole grain flours presented higher TO-AX content, while WE-AX content was higher in durum wheat samples in comparison to bread wheat samples. 

### 3.2. Total Phenols Content

Total phenols content in analyzed samples ranged from 5.44 to 26 mg GAE/g extract in Aldura white flour and Marius whole grain flour, respectively. It is observed that within whole grain samples, the flour corresponding to Marius variety exhibited a significantly higher content (*p* < 0.05) of total phenols in comparison to the rest of the whole grain flours.

### 3.3. Tocopherols

**Figure 1:**
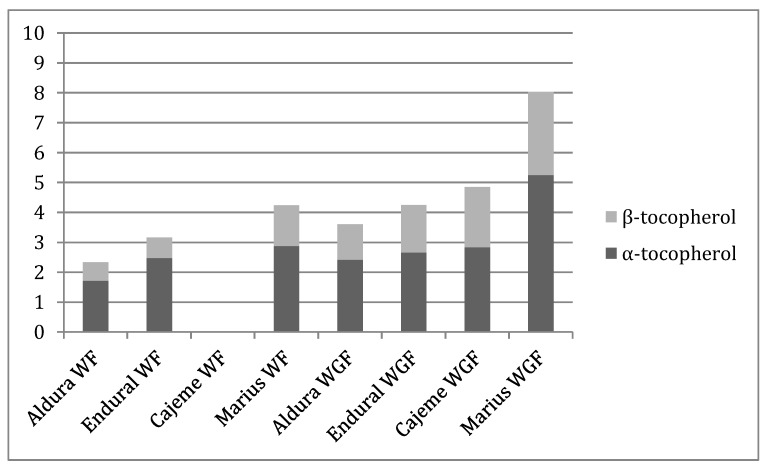
α- and β-tocopherol contents (μg/g flour, dry weight) of different wheat flours. WF: white flour; WGF: whole grain flour.

In present study, total tocopherols content (Table 2, Figure 1) ranged from 2.34 μg/g in Aldura white flour to 8.02 μg/g in Marius whole grain flour. As expected, tocopherols content was significantly higher (*p* < 0.05) in all whole wheat flour in comparison with white wheat flour. α-, β- and γ-Tocopherols were identified in all samples analyzed, except in the case of Cajeme white flour, in which no tocopherols were characterized. In all cases, α-tocopherol was the major isoform. Comparing durum wheat flour varieties with bread wheat flour varieties, the present study showed that the content of total tocopherols in whole grain flour was highest (*p* < 0.05) in bread wheat varieties, mainly due to the highest amount in α- and β-tocopherols. It is also observed that in the case of the whole grain flours, Endural and Marius samples present a significantly higher amount than Aldura variety and Cajeme variety (comparing their correspondent durum and bread wheat varieties).

### 3.4. Evaluation of Antioxidant Activity

In the present study, the antioxidant activity of wheat flours was evaluated using three different in vitro assays. Total antioxidant activity was measured using DPPH and reducing power assays while, the capacity of inhibiting lipid peroxidation was evaluated through the β-carotene/linoleate assay. The antioxidant activity, determined by DPPH assay, showed higher values (higher EC_50_ means lower antioxidant activity) than all the other methods for all whole flour and white flour samples (Table 2). All studied whole flours presented significantly higher (*p* < 0.05) antioxidant activity (lower EC_50_) than white flours obtained from the same wheat variety. These results could be explained due to the higher antioxidant compounds content (e.g., total phenols and tocopherols) presents in whole grain flour in comparison to their corresponding white flours. According to the obtained results, all whole grain flours from bread wheat varieties present significantly lower (*p* < 0.05) EC_50_ values than durum wheat flours. According to the obtained results, all whole grain flours display better antioxidant properties than white flours from all antioxidant assays, except in the case of Endural sample. Comparing bread and durum wheat varieties, whole grain flours from bread varieties (Cajeme and Marius) showed better antioxidant activity (lower EC_50_ values). In general, Marius whole grain flour presented the lowest EC_50_ values in comparison to other whole grain samples. This means that Marius whole grain flour is the sample with better antioxidant properties, and this is in accordance with the content of total phenols and tocopherols found in this sample (Table 2). Comparing white flours samples, durum wheat varieties showed better capacity of inhibiting lipid peroxidation (lower EC_50_ values in β-carotene/linoleate assay) than bread wheat varieties. These differences are perhaps due to the higher contents of lutein in durum wheat.

## 4. Discussion

### 4.1. Dietary Fiber and Arabinoxylans

Cereals, and particularly wheat grains, are an important source of dietary fiber (DF), which is known to be beneficial against several diseases, including colon cancer, diabetes, etc. [11].

According to Regulation (EC) No 1924/2006 [12] the claim ‘source of fiber’ may be used for white flours analyzed in the present study as their content in total dietary fiber (TDF) is higher than 3 g per 100 g. Due to TDF content in whole grain flours and bran fractions analyzed is higher than 6 g per 100 g, these samples may use the claim ‘high fiber’. Dietary fiber plays an important role in the gastrointestinal function and in this sense, the analyzed bran fractions met the conditions for use the following health claims (samples contain at least 6 g of fiber per 100 g) according to Regulation (EU) No 432/2012 [13] and Regulation (EC) No 1924/2006 [12]: “Wheat bran fiber contributes to an acceleration of intestinal transit” and “Wheat bran fiber contributes to an increase in fecal bulk”. 

Since the term ‘functional food’ was first introduced in Japan in 1984, numerous definitions of that have been given by different authors [14]. According to EU Project ‘Functional Food Science in Europe’ (FUFOSE), functional food is defined as ‘Foods that are satisfactorily demonstrated to affect beneficially one or more target functions in the body, beyond adequate nutritional effects, in a way that is relevant to either an improved state of health and well-being and/or reduction of risk of disease. Functional foods must remain foods and they must demonstrate their effects in amounts that can normally be expected to be consumed in the diet: they are not pills or capsules, but part of a normal food pattern’ [15]. In accordance to this definition and regarding the dietary fiber content, the bran fractions analyzed could be considered such as functional foods as they are foods that have demonstrated a beneficial effect related to gastrointestinal health.

Comparing the obtained values of TDF with those obtained by other authors, lower TDF results were reported by Rainakari et al. (2016) [16] in different whole grain bread wheat flours (with values around 10.2 to 15.7 g/100 g, dw) and similar results were reported in durum-type wheat whole grains (12.7 to 20.0 g/100 g, dw) by Marotti et al. (2012) [17]. In the case of bran, similar TDF results were reported by Sobota et al., (2015) [18] and Schmiele et al., (2012) [19] with values of 28.8 g/100 g and 43.4 g/100 g, respectively. Moreover, as part of the HEALTHGRAIN program, the extent of variation in the content of dietary fiber of different wheat types and varieties was investigated. The results of this study showed that the mean value of total dietary fiber in 150 bread wheat lines was 15.1 g/100 g [20], which is in accordance with the results obtained in the present study (the mean value of TDF in analyzed whole grain flour was around 16.8 g/100 g, dw).

Regarding different fractions of dietary fiber (insoluble and soluble dietary fiber) and comparing the obtained results with those found by other authors, Marotti et al. (2012) [17] reported a similar proportion of IDF (85% of TDF), while Gélinas and McKinnon (2013) [21] reported a lower percentage (71–75% of TDF) in different wheat cultivars comparing with our results. Regarding SDF content, Marotti et al. (2012) [17] reported similar amounts in different durum-type wheat whole grains (1.8 to 3.7 g/100 g, dw). In the case of bran, Sobota et al. (2015) [18] found a content of 23.7 and 5.1 g/100 g dw, corresponding to IDF and SDF, respectively. Taking into account that the fiber Recommended Dietary Intake (RDI) is 25 g/day [22], the consumption of 100 g of the analyzed whole grain wheat flours could cover more than 52.4% of the RDI of this macronutrient, as in the case of Aldura variety.

Arabinoxylans (AX) are one of the major components of non-starch polysaccharides (NSP) in wheat grains, representing around 70% of the NSP. These compounds are mainly found in wheat starchy endosperm and white flour fractions [17,23]. AX are hemicelluloses that have a xylose backbone with arabinose side chains and occurring mostly in the bran and aleurone fractions, which are not distributed uniformly in the wheat kernel (20%–27% of the aleurone, 23%–32% of the bran and 2%–4% of the endosperm). Taking into account the solubility of these compounds, it can be classified into water-extractable (WE-AX) and water-unextractable (WU-AX). AX content in wheat flour is relatively low, but this polymer plays an important role in flour functionality (WE-AX and WU-AX have different effects on breadmaking) [11].

These compounds have several and important health benefits, such as prebiotic effect and antioxidant ability, which could explain that AX are prevention agents of colon cancer diseases. At the metabolic level, AX contributes to glycemic and cholesterol levels control, being also considered as an immunoregulator agents [24].

In particular, AX produced from wheat endosperm have approved the following health claim according to Regulation (EU) No 432/2012 [13]: “Consumption of AX as part of a meal contributes to a reduction of the blood glucose rise after that meal”. This claim may be used only for food, which contains at least 8 g of AX-rich fiber produced from wheat endosperm (at least 60% AX by weight) per 100 g of available carbohydrates in a quantified portion as part of the meal. In this sense, samples D-E#2-WF, S-C#1-WF and S-M#1-WF met the stipulated conditions for use the referred claim and could be considered as functional foods. It is important to underline that the only permitted reduction of disease risk claim related to arabinoxylans has been approved for arabinoxylan produced from wheat endosperm. In this sense, wheat flours have potential properties to be used as functional ingredients in the development of different food products with improved quality. 

Comparing with other authors results, Vignola et al. (2016) [11] analyzed TO-AX and WE-AX content on whole grain and white flour of eleven wheat varieties, and reported that TO-AX content ranged between 7.59–12.80 g/100 g and 4.43–5.65 g/100 g in whole grain flour and white flour, respectively. In the same study, WE-AX content was higher in white flour (0.33–0.75 g/100 g) than in whole grain (0.35–0.72 g/100 g), probably due to reduced accessibility of the bran fraction to water, as was also observed in Cajeme variety samples. Turner et al., (2008) [25] reported, that the amount of WE-AX is especially important in durum wheat flours, which is used for pasta production, and high values of WE-AX may improve the pasta quality, due to the significant positive influence on cooked pasta resilience and stickness of these compounds (WE-AX increase water absorption and reduce dough strength). In this sense, the analyzed durum whole grain flours showed higher amount of WE-AX than bread flours and therefore presented better qualities for pasta production (Table 1).

Marotti et al. (2012) [17] studied ten durum-type wheat varieties and reported AX content of whole grains (average value of 3.27 g/100 g dw) lower than the ones reported in the present study. Moreover, in HEALTHGRAIN program, AX was also analyzed and the authors reported a mean value of 1.93% and 0.51% of flour corresponding to TO-AX and WE-AX, respectively [20], being WE-AX amount in accordance with our results. Comparing the obtained results for bran fraction, Gebruers et al. (2008) [26] reported higher values for TO-AX (8.9%–18.0%) and similar values for WE-AX content (0.3–0.55%) in bran fraction.

Vignola et al. (2016) [11] reported that the harvesting year is one of the main responsible factors for the variability found in TO-AX content of wheat flours, while WE-AX content was higher influenced by genotype. This statement was also observed in the analyzed samples, since TO-AX content was significantly higher (*p* < 0.05) in samples of whole grain flour harvested in the second year.

### 4.2. Total Phenols Content

Phenolic acids are not evenly distributed in wheat, and wheat bran presents a high concentration of these compounds, including vanillic acid, *p*-coumaric acid and, largely, ferulic acid [27]. During milling process brand and germ are lost, resulting in lower vitamin, mineral, phenolic compounds, and fiber content. For this reason, it is recommended that half of the grains consumed are whole grains. Hence whole grains flours should be promoted against refined flours [6].

It is known that total phenolics might be the major contributor of wheat antioxidant activities, since these compounds are highly correlated with this bioactivity [1]. The phenolic acids in wheat grains are often presenting the bound form with other grain components such as cellulose, glucan, pentosane and starch. The highest concentration of flavonoids and phenolic acids is located in the aleurone layer of cereal grains, but these compounds are also found in lower amount in the embryos and seed coat of grains [28,29]. Particularly, Beta et al. (2005) [30] observed that phenolic compounds were concentrated in pearled fractions representing ≤20% of the outer layers of wheat. In accordance with this observation, the results of the present study showed that whole grain flours had higher amounts of total phenols than white flours in all the analyzed varieties (Table 2). It is known that both biotic and abiotic factors, including environmental and agronomic conditions, could influence the biosynthetic pathway of phenolic compounds and, as consequence, their content on wheat varieties may vary. Moreover, it is important to take into account that the genetic-environmental interactions, could result in a large phenolic content (among others) fluctuation in cereal species and cultivars of the same species [31]. Therefore, for future studies, the agronomic and environmental influence for the selected varieties will be evaluated.

Our results were in concordance with those reported by Kosík et al. (2014) [29] that evaluated free phenols content and antioxidant activity of winter wheat (*Triticum aestivum* L.) white flour, whole grain flour and bran fraction in ecological and integrated farming system. Kosík et al. (2014) [29] reported that free phenolic content was also higher in the whole flour than in the white flour (185.6 µg GAE/g and 97.0 µg GAE/g respectively). Moreover, Vaher et al. (2010) [28] analyzed total phenolic content in wheat grains, their respective bran fraction and flour of different winter and spring varieties. In this work, the total phenolic content (determined by the Folin-Ciocalteu assay) of the bran layer was the highest (1258–3157 μg/g), compared to the content of grains (168–459 μg/g) and flour (44–140 μg/g).

### 4.3. Tocopherols

Vitamin E is an important lipophilic antioxidant bioactive compound present in food and the term is used to describe a family of eight lipid-soluble antioxidants (tocochromanols) with two types of structures, four designated tocopherols (α, β, γ and δ-tocopherol) and the other four designated tocotrienols (α, β, γ and δ -tocotrienol) [32]. Tocopherols and tocotrienols present a free hydroxyl group which stabilize free radicals and stop the propagation phase of the oxidation chain reaction, being responsible for the antioxidant activity of vitamin E. Apart from their antioxidant properties, tocopherols content of cereals are related to human health benefits as these compounds could modulate degenerative diseases like cardiovascular diseases or cancer [33].

The major isoforms in wheat grains are α-and β-tocopherol [32]. It is known that milling process could negatively affect vitamin E content, decreasing its content in white flours [34]. In wheat grain, the germ fraction and aleurone tissue are very rich in tocopherols, therefore the refining process of wheat grain results in a substantial loss of this bioactive compound [2,35].

The results obtained in the present study were in accordance with those reported by Sedej et al. (2010) [35] that evaluated the tocopherols content in wheat flours, being total tocopherols content in whole wheat flour higher than light wheat flour, and α–tocopherol was also the major isoform in their wheat analyzed flours. Moreover, Engelsen and Hansen (2009) [2] analyzed vitamin E content in different wheat milling fractions from roller milling and reported that wheat flour contains 7.8 μg/g of α–tocopherol and 4.6 μg/g of β–tocopherol, being these values higher than obtained in the present study. While, Lv et al. (2012) [1] evaluated ten bread red winter wheat (*Triticum aestivum* L.) varieties (SS520, SSMPV57, SS5205, USG3555, USG3665, USG3315, Branson, Shirley, Jamestown and Chesapeake) and reported that α–tocopherol content in white flour ranged from 0.3 to 0.59 μg/g, being these amounts much lower than found in our study.

Cereal grains are also relevant sources of tocotrienols in the human diet. These compounds are mainly located in bran and endosperm fractions. Among tocotrienols, β-tocotrienol is the prevailing isoform in wheat [36,37,38]. It is reported that the content of α- and β-tocotrienol in wheat (*T. aestivum* L.) is 2.83 and 16.54 μg/g, respectively [36]. Similar results have been obtained by Lachman et al. (2018) [37], who studied different wheat (*T. aestivum* L.) genotypes and reported values of 1.17–3.58 and 4.72–16.16 μg/g, corresponding to α- and β-tocotrienol, respectively. As part of HEALTGRAIN Program, the tocol composition, including tocopherols and tocotrienols, of 26 genotypes of wheat (*Triticum aestivum* var. aestivum) has been studied [38]. In which, the content of α- and β-tocotrienol ranged between 3.2–9.7 and 9.8–50.2 μg/g, respectively. In general, both genetic and environmental factors have an important impact on the concentration of tocopherols and tocotrienols in different wheat genotypes. In this sense, it has been observed that the genotype and the harvesting year have significant effects on total tocol and tocotrienol content, whereas the content of tocopherols is only influenced by the wheat genotype [38].

In addition to vitamin E (tocopherols and tocotrienols), wheat also contains other lipophilic antioxidant compounds, namely carotenoids. They are natural pigments characterized that can be classified in two main types of compounds, namely carotenes (lycopene and β-carotene) and xantophylls (zeaxanthin and lutein) [39]. It is important to take into account that the content of carotenoids in plant foods varies depending on the cultivar, the geographical origin and the environmental factors, it has been suggested that high temperatures and drought during the growing season promoted carotenoid biosynthesis in cereal grains [40,41]. Moreover, carotenoids are heterogeneously distributed in the wheat grain, being the endosperm the highest in lutein, whereas the outer layers of the kernel are richer in zeaxanthin and β-carotene [42].

Bread wheat (*Triticum aestivum* L.) is widely used for bakery products elaboration and its content of carotenoids is usually lower, comparing to durum wheat (*Triticum turgidum* L.), which is characterized by its yellow color (attributed to the high carotenoid content) and it is used for pasta production [43]. The content of carotenoids in wheat has been studied by different authors and it has been observed that lutein is the main carotenoid in this cereal (80%–90% of total carotenoids), while zeaxanthin, α-carotene, β-cryptoxanthin, and β-carotene are found in lower amounts [42]. 

Okarter et al. (2010) [44] studied the phytochemical content of six diverse varieties of whole wheat (bread and durum wheat) and their results showed that total carotenoid content ranged from 1.48 to 2.71 μg/g. In this study the predominant carotenoid found was lutein (0.67–2.11 μg/g), while zeaxanthin (0.25–0.53 μg/g), β-carotene (0.18–0.36 μg/g), and β-cryptoxanthin (0.12–0.20 μg/g) were detected in lower amounts. Moreover, α-carotene was not found in any analyzed wheat varieties. This pattern was also observed by Mamatha et al. (2011) [40] in bread wheat (*T. aestivum*). These authors reported values of 0.84–0.86 μg/g, 0.73–0.77 μg/g, 0.002–0.003 μg/g and 0.06–0.13 μg/g for total carotenoids, lutein, zeaxanthin and β-carotene, respectively. Furthermore, Lv et al. (2012) [1] compared the phytochemical composition of ten different wheat (*Triticum aestivum* L.) varieties grown in Maryland and reported that the content of lutein and zeaxanthin ranged from 0.27–0.46 and 0.08–0.13 μg/g, respectively. However, these authors did not find β-carotene in any of the analyzed flour samples. Whent et al. (2012) [45] reported higher values of lutein (1.5–4.0 μg/g) in different whole wheat flour from five spring wheat cultivars. 

Although carotenoids are generally minor components in wheat grains, due to the high consumption of wheat-based foods, this cereal constitute an important source of carotenoids in most world regions [43].

### 4.4. Evaluation of Antioxidant Activity

The antioxidant capacity of wheat grain fractions is mainly associated to the aleurone content, which can be related to significant amounts of hydroxycinnamic acids, mainly ferulic acid. Antioxidant activity of extracts obtained from wheat bran fraction reveals a stronger potential than other wheat fractions [27]. Antioxidants achieve beneficial effects on health through different mechanisms, such as directly reacting with free radicals mitigating its effects, chelating transition metals, reducing peroxides, and promoting the antioxidative defense enzyme system [27].

Components of whole grains, whether in their native and processed states, have a similar antioxidant capacity, as do many fruits and vegetables. However, the antioxidant capacity of refined grains (e.g., white flours) is much reduced in comparison to their correspondent whole grains (about 20% of the antioxidant capacity of whole grains) [6]. There are several studies in bread wheat flours that show that whole wheat flour has higher antioxidant capacity than white wheat flour. Particularly, Yu et al. (2013) [46] studied the antioxidant properties of refined and whole wheat flour and demonstrated that the antioxidant activity was higher in whole wheat flours compared to refined flours. Moreover, Kosík et al. (2014) [29] evaluated the antioxidant activity of winter wheat (*Triticum aestivum* L.) by the DPPH assay, and reported that the antioxidant potential of whole grain flour was significantly higher than in the white flour. Sedej et al. (2010) [34] obtained similar results that confirm that whole grain flour has better antioxidant properties than light wheat flour.

## 5. Conclusions

Wheat is an important agricultural cereal and a worldwide primary food ingredient, which contains considerable beneficial nutritional components. The analyzed varieties could be considered a significant source of dietary fiber, being insoluble dietary fiber the major fraction representing up to 96% of total dietary fiber. In the case of white flours, it was observed that samples corresponding to durum wheat varieties (Endural and Aldura) were significantly higher (*p* < 0.05) in total dietary fiber content, than flours from bread wheat varieties (Cajeme and Marius). Arabinoxylans are the main components of non-starch polysaccharides in wheat grain. In the particular case of WE-AX, the content of these compounds was generally higher in flour obtained from durum wheat varieties. This fact has an important technological repercussion because WE-AX improves the quality of pasta products obtained from durum wheat flours. Both dietary fiber and arabinoxylans are important compounds from a nutritional and human health point of view. In this sense, Regulation (EU) No 432/2012 establishing three permitted health claims made on wheat (two of them are related to wheat bran fiber and the third one is related to arabinoxylans produced from wheat endosperm). In the present study, the analyzed samples met the stipulated conditions for using all these claims, supporting the possibility to consider the studied wheat flours (especially whole flour and bran fraction) as functional ingredients or functional foods with approved nutritional and health claims according to the European regulation.

Moreover, as far as we know, this is the first study in which the analyzed wheat varieties were characterized in terms of other phytochemical compounds, which are interesting due to their antioxidant properties, such as total phenolic compounds and tocopherols, as well as their individual vitamers. All whole flour demonstrated higher amounts of total phenols and total tocopherols, than their corresponding white flour. α-Tocopherol was the major isoform found in the studied samples, being higher in whole grain flour (*p* < 0.05) than in white wheat flours. Moreover, whole grain flours obtained from bread wheat varieties display better antioxidants properties than whole grain flour from durum wheat. Due to their phytochemical composition, analyzed wheat varieties are beneficial foods for health, whose consumption and, particularly, the consumption of whole wheat flours, must be promoted.

## Figures and Tables

**Table 1:** Dietary fiber (insoluble, soluble and total) and arabinoxylans (water soluble and total) contents in different bread and durum wheat flours (g/100 g, dry weight) (mean ± SD).

	**White Flour**							
	**Wheat Variety**	**Year**	**Sample Code**	**IDF**	**SDF**	**TDF**	**WE-AX**	**TO-AX**
***Triticum turgidum* L.**	**Aldura**	**1**	D-A#1-WF	5.5 ± 0.3 ^b,c,B^	1.0 ± 0.0 ^d,A^	7.0 ± 0.4 ^d,B^	1.0 ± 0.1 ^e,B^	6.0 ± 0.2 ^c,B^
		**2**	D-A#2-WF	4.9. ± 0.4 ^a,b.A^	0.9. ± 0.0 ^d,A^	5.8. ± 0.5 ^b,c,A^	0.5. ± 0.0 ^c,A^	4.2. ± 0.1 ^a,A^
	**Endural**	**1**	D-E#1-WF *	-	-	-	-	-
		**2**	D-E#2-WF	4.4 ± 0.3 ^a^	1.2 ± 0.0 ^e^	5.4 ± 0.4 ^a,b,c^	0.6 ± 0.0 ^c^	6.8 ± 0.5 ^d,e^
***Triticum aestivum* L.**	**Cajeme**	**1**	S-C#1-WF	6.5 ± 0.1 ^d,B^	0.4 ± 0.1 ^c,A^	6.9 ± 0.1 ^d,B^	0.5 ± 0.0 ^c,A^	6.5 ± 0.6 ^c,d,e,A^
		**2**	S-C#2-WF	5.8 ± 0.5 ^c,d,A^	0.4 ± 0.0 ^c,A^	6.2 ± 0.5 ^c,d,A^	0.9 ± 0.0 ^d,B^	6.3 ± 0.1 ^c,d,A^
	**Marius**	**1**	S-M#1-WF	4.5 ± 0.2 ^a,A^	0.1 ± 0.0 ^a,A^	4.7 ± 0.3 ^a,A^	0.3 ± 0.0 ^a,A^	7.0 ± 0.4 ^e,B^
		**2**	S-M#2-WF	4.7 ± 0.2 ^a,b,A^	0.3 ± 0.0 ^b,B^	5.0 ± 0.2 ^a,b,A^	0.4 ± 0.0 ^b,B^	4.9 ± 0.2 ^b,A^
	**Whole Grain Flour**							
	**Wheat Variety**	**Year**	**Sample Code**	**IDF**	**SDF**	**TDF**	**WE-AX**	**TO-AX**
***Triticum turgidum* L.**	**Aldura**	**1**	D-A#1-WGF	12.1 ± 0.3 ^a,A^	1.5 ± 0.1 ^a,b,c,B^	13.1 ± 0.3 ^a,A^	1.1 ± 0.0 ^d,B^	6.7 ± 0.1 ^a,A^
		**2**	D-A#2-WGF	12.4 ± 1.0 ^a,A^	0.9 ± 0.0 ^a,b,A^	13.3 ± 1.0 ^a,A^	0.8 ± 0.1 ^b,A^	8.1 ± 0.3 ^c,B^
	**Endural**	**1**	D-E#1-WGF	15.9 ± 0.8 ^b,B^	1.8 ± 0.3 ^c,A^	17.0 ± 1.0 ^b,c,B^	1.1 ± 0.0 ^d,A^	6.8 ± 0.2 ^a,A^
		**2**	D-E#2-WGF	13.1 ± 1 ^a,A^	3.2 ± 0.4 ^d,B^	16.3 ± 1 ^b,A^	1.1 ± 0.1 ^d,A^	8.2 ± 0.3 ^c,B^
***Triticum aestivum* L.**	**Cajeme**	**1**	S-C#1-WGF	18.3 ± 0.3 ^b,A^	1.4 ± 0.1 ^a,b,c,A^	19.7 ± 0.3 ^d,A^	0.9 ± 0.0 ^c,B^	7.3 ± 0.4 ^b,A^
		**2**	S-C#2-WGF	17.4 ± 1.5 ^b,A^	1.6 ± 0.0 ^b,c,A^	19.8 ± 1.8 ^c,d,A^	0.7 ± 0.1 ^b,A^	8.5 ± 0.4 ^c,B^
	**Marius**	**1**	S-M#1-WGF	17.6 ± 0.4 ^b,A^	0.8 ± 0.1 ^a,A^	18.4 ± 0.4 ^b,c,d,A^	0.6 ± 0.0 ^a,A^	6.7 ± 0.7 ^a,A^
		**2**	S-M#2-WGF	17.2 ± 0.6 ^b,A^	1.0 ± 0.1 ^a,b,B^	18.2 ± 0.7 ^b,c,d,A^	0.5 ± 0.0 ^a,A^	6.5 ± 0.4 ^a,A^
	**Bran**							
	**Wheat Variety**	**Year**	**Sample Code**	**IDF**	**SDF**	**TDF**	**WE-AX**	**TO-AX**
***Triticum turgidum* L.**	**Aldura**	**1**	D-A#1- Bran	23.4 ± 1.3 ^a,A^	2.5 ± 0.2 ^b,B^	23.5 ± 1.3 ^a,A^	1.1 ± 0.1 ^c,A^	7.7 ± 0.3 ^a,b,A^
		**2**	D-A#2- Bran	22.8 ± 2.0 ^a,A^	0.8 ± 0.1 ^a,A^	23.6 ± 2.0 ^a,A^	1.1 ± 0.1 ^c,A^	13.5 ± 0.4 ^e,B^
	**Endural**	**1**	D-E#1- Bran *	-	-	-	-	-
		**2**	D-E#2- Bran	25.7 ± 1.7 ^a^	6.5 ± 0.3 ^e^	32.1 ± 2.0 ^b^	1.9 ± 0.1 ^e^	10.6 ± 0.6 ^d^
***Triticum aestivum* L.**	**Cajeme**	**1**	S-C#1- Bran	43.2 ± 1.0 ^b,A^	3.3 ± 0.3 ^c,A^	46.8 ± 1.0 ^c,A^	1.7 ± 0.1 ^d,B^	8.4 ± 1.3 ^b,c,A^
		**2**	S-C#2- Bran	47.4 ± 2.1 ^c,B^	4.2 ± 0.1 ^d,B^	51.6 ± 2.1 ^d,B^	0.3 ± 0.0 ^a,A^	13.8 ± 1.0 ^e,B^
	**Marius**	**1**	S-M#1- Bran	44.1 ± 2.0 ^b,c,A^	2.1 ± 0.1 ^b,A^	46.1 ± 1.6 ^c,A^	1.2 ± 0.1 ^c,B^	6.3 ± 0.6 ª^,A^
		**2**	S-M#2- Bran	45.0 ± 2.2 ^b,c,A^	2.4 ± 0.2 ^b,A^	47.4 ± 2.2 ^c,A^	0.9 ± 0.0 ^b,A^	9.5 ± 0.9 ^c,d,B^

In each column, different letters mean statistically significant differences (*p* < 0.05) compared by Tukey test; small superscript letter (a–e) means differences between all white or whole grain analyzed flours, whereas capital superscript letter (A,B) means difference due to the harvesting year for the same wheat variety. IDF: Insoluble Dietary Fiber; SDF: Soluble Dietary Fiber; TDF: Total Dietary Fiber; WE-AX: Water-Extractable Arabinoxylans; TO-AX: Total Arabinoxylans. * Due to the sample D-E#1-WF was no available, results corresponding to samples D-E#1-WF and D-E#1- Bran are not shown.

**Table 2:** Total phenols content (mg GAE/g extract), total tocopherol content (μg/g flour, dry weight) and antioxidant activity (EC_50_, mg/mL methanolic extract) of different bread and durum wheat flours (mean ± SD).

		**White Flour**						
	**Wheat Variety**	**Total Phenols**	**α-Tocopherol**	**β-Tocopherol**	**Total Tocopherols**	**DPPH**	**Reducing Power**	**β-Carotene Bleaching Inhibition**
***Triticum turgidum* L.**	**Aldura**	5.44 ± 0.09 ^a^	1.72 ± 0.01 ^a^	0.62 ± 0.02 ^a^	2.34 ± 0.01 ^a^	17.4 ± 0.4 ^c^	2.98 ± 0.03 ^c^	1.71 ± 0.02 ^a^
	**Endural**	14.6 ± 0.4 ^c^	2.48 ± 0.02 ^b^	0.68 ± 0.00 ^b^	3.16 ± 0.03 ^b^	14.5 ± 0.4 ^b^	2.00 ± 0.01 ^a^	2.01 ± 0.09 ^b^
***Triticum aestivum* L.**	**Cajeme**	14.2 ± 0.5 ^c^	nd	nd	nd	19.5 ± 0.2 ^d^	2.26 ± 0.11 ^b^	4.63 ± 0.06 ^c^
	**Marius**	11.1 ± 0.5 ^b^	2.88 ± 0.01 ^c^	1.36 ± 0.01 ^c^	4.24 ± 0.01 ^c^	11.5 ± 0.5 ^a^	3.9 ± 0.1 ^d^	5.7 ± 0.2 ^d^
		**Whole Grain Flour**						
	**Wheat Variety**	**Total Phenols**	**α-Tocopherol**	**β-Tocopherol**	**Total Tocopherols**	**DPPH**	**Reducing Power**	**β-Carotene Bleaching Inhibition**
***Triticum turgidum* L.**	**Aldura**	20.7 ± 0.5 ^a^	2.42 ± 0.05 ^a^	1.19 ± 0.08 ^a^	3.63 ± 0.03 ^a^	8.3 ± 0.1 ^c^	2.59 ± 0.06 ^d^	1.80 ± 0.07 ^d^
	**Endural**	20 ± 1 ^a^	2.66 ± 0.05 ^b^	1.59 ± 0.03 ^b^	4.26 ± 0.08 ^b^	9.13. ± 0.08 ^d^	2.33. ± 0.07 ^c^	1.63. ± 0.04 ^c^
***Triticum aestivum* L.**	**Cajeme**	20.0 ± 0.9 ^a^	2.84 ± 0.03 ^c^	2.01 ± 0.02 ^c^	4.85 ± 0.01 ^c^	6.1 ± 0.2 ^b^	0.9 ± 0.2 ^a^	1.30 ± 0.02 ^b^
	**Marius**	26 ± 1 ^b^	5.25 ± 0.05 ^d^	2.78 ± 0.04 ^d^	8.02 ± 0.01 ^d^	4.19 ± 0.05 ^a^	1.57 ± 0.02 ^b^	1.13 ± 0.07 ^a^

In each column, different letters (a–d) mean statistically significant differences (*p* < 0.05) compared by Tukey test. nd (non-detected).
